# The Preparation and Properties of Nanocomposite from Bio-Based Polyurethane and Graphene Oxide for Gas Separation

**DOI:** 10.3390/nano9010015

**Published:** 2018-12-23

**Authors:** Yongsheng Zhang, Jun Ma, Yao Bai, Youwei Wen, Na Zhao, Xiaoling Zhang, Yatao Zhang, Qian Li, Liuhe Wei

**Affiliations:** 1 School of Chemical Engineering and Energy, Zhengzhou University, Zhengzhou 45001, China; yzhang@zzu.edu.cn (Y.Z.); 18838959658@163.com (J.M.); 18839781013@163.com (Y.B.); Wyw1426969592@163.com (Y.W.); m18801175531@163.com (X.Z.); 2 National Center for International Research of Micro-nano Molding Technology, Zhengzhou University, Zhengzhou 450001, China; anna@zzu.edu.cn; 3 College of Chemistry and Molecular Engineering, Zhengzhou University, Zhengzhou 45001, China; weiliuhe@zzu.edu.cn

**Keywords:** bio-based polyurethane, nanocomposite, membrane, gas separation

## Abstract

Petroleum depletion and climate change have inspired research on bio-based polymers and CO_2_ capture. Tung-oil-based polyols were applied to partially replace polyether-type polyols from petroleum for sustainable polyurethane. Tung-oil-based polyurethane (TBPU), was prepared via a two-step polycondensation, that is, bulk prepolymerization and chain extension reaction. The graphene oxide (GO) was prepared via Hummer’s method. Then, TBPU was composited with the GO at different ratios to form a TBPU/GO hybrid film. The GO/TBPU films were characterized by fourier transform infrared spectroscopy (FTIR), differential scanning calorimeter (DSC), thermal gravimetric analysis (TGA) and scanning electron microscope (SEM), followed by the measurement of mechanical properties and gas permeability. The results showed that the addition of tung-oil-based polyols enhanced the glass transition temperature and thermal stability of TBPU. The mechanical properties of the hybrid film were significantly improved, and the tensile strength and elongation at break were twice as high as those of the bulk TBPU film. When the GO content was higher than 2.0%, a brittle fracture appeared in the cross section of hybrid film. The increase of GO content in hybrid films improved the selectivity of CO_2_/N_2_ separation. When the GO content was higher than 0.35%, the resulting GO agglomeration constrained the gas separation and permeation properties.

## 1. Introduction

Growing concerns over climate changes and the desire to reduce the dependence on crude oil have intensified worldwide interest in green chemicals and materials from renewable biomass [1,2]. Furthermore, continuous attention has been paid to the greenhouse effect caused by CO_2_ emissions owing to the increasing use of fossil fuels. The capture and removal of CO_2_ from gas mixture has gained worldwide attention [3]. Membrane separation of CO_2_ is an energy-saving technology and is advantageous because of its simple device, low cost and high efficiency. The materials for CO_2_ separation mainly include polymeric membrane, molecular sieve membrane, facilitated transport membrane, and organic-inorganic hybrid membrane. The membrane material can be determined based on the specific different separation process [4]. Polymeric materials have been frequently used in this area, and the separation principle was mainly based on the solution–diffusion mechanism. Among the polymeric membranes, bio-based polyurethane, comprising soft and hard segments with a sustainable component, is of particular interest. The permeability of gas molecules could be enhanced by hard segments and soft segments, which are important for selectivity. Therefore, the CO_2_ permeate flux and the selective separation effect could be influenced by soft and hard segment ratios [5].

Polyurethane (PU) is prepared by condensation polymerization between polyether- or polyolefin-type polyols and isocyanates, together with chain extenders. PU contains urethane bonds and alternating soft and hard connecting segments [6]. Many researchers have sought alternatives to polyols to prepare sustainable PU. Among them, bio-based polyurethane synthesized using vegetable-oil-based polyol has been a hot research topic [7]. Tung oil, derived from the tung tree, a species of *Vernicia* in the spurge family native to Southern China, can be transformed into polyol which is attractive for its hydroxyl groups. The greatest challenge of the use of polyurethane in gas separation is the low selectivity and the low resistance to plasticization. Graphene oxide (GO) is a promising candidate for organic–inorganic hybrid membranes, with good gas capture and selectivity characteristics derived from its single-layer and defective structure. There have been several approaches to preparing GO/PU hybrid membranes: solution blending, in-situ polymerization, and melt-blending polymerization.

Nevertheless, the compatibility between hydrophilic GO and hydrophobic polyurethane is still challenging. Therefore, GO is generally chemically modified to adapt to the organic matrix [8]. Functionalized graphene layers produced from graphite oxide via thermal reduction or isocyanate treatments were used to strengthen thermoplastic polyurethane by Kim et al [9]. They found the use of solution-blending more effective than melt-blending polymerization to get well-distributed nanocomposites [9]. By changing the quality of KMnO_4_, a group of GO with different degrees of oxidation was obtained by Wang et al. [10] through the improved Hummer’s method. Then GO/PU composite membranes with different oxidation levels and quantities of GO were prepared via in-situ polymerization, followed by an investigation of the CO_2_/N_2_ selectivity and morphology characterization. The results showed that hybrid membranes containing well-distributed GO exhibited enhanced permeability and selectivity for CO_2_. However, surface defects of the GO sheet increased if it was overoxidized, and the properties of the hybrid membrane were constrained, accompanied by GO aggregation at a high quantity.

In the present work, the petrochemically derived polyether polyol was partially replaced by tung-oil-based polyol. Tung-oil-based polyurethane (TBPU) was prepared by two-step condensation polymerization using tung-oil-based polyol and isocyanate assisted by chain extension. Then hybrid membranes were prepared by incorporating GO into TBPU. GO was made via Hummer’s method, followed by blending with TBPU at a certain proportion, and then GO/TBPU hybrid membrane was formed when the solvent was vaporized. The structure and properties of TBPU and the hybrid membrane were characterized by fourier transform infrared spectroscopy (FTIR), differential scanning calorimeter (DSC), scanning electron microscope (SEM), thermal gravimetric analysis (TGA), and then the GO/TBPU nanocomposite membranes were investigated for their mechanical properties, gas permeability, and selectivity. GO/TBPU nanocomposite is believed to be a promising material in hybrid membrane applications.

## 2. Materials and Methods

### 2.1. Materials

Diphenylmethane diisocyanate (MDI), polyethylene glycol (PEG), and 1,4-Butanediol (BDO) were acquired from Shanghai Jinchuan Reagent Co., Ltd. (Shanghai, China). The PEG and BDO were completely dried at 80 °C under vacuum pressure before use. The tung-oil-based polyols were prepared via hydroxylation by Prof. Zhiyong Ren from the Institute of Chemistry, Henan Academy of Sciences (Zhengzhou, China), and used as received [11]. Natural graphite powders (ca. 45 mm) was purchased from Sinopharm Chemical Regent (Shanghai, China). Concentrated sulfuric acid (H_2_SO_4_, 98 wt %), phosphoric acid (H_3_PO_4_, 85 wt %), hydrochloric acid (HCl) and hydrogen peroxide (H_2_O_2_, 30 wt %) were all obtained from Tianjin Fengchuan Chemical Reagent Technologies Co., Ltd. (Tianjin, China). Potassium permanganate (KMnO_4_) was purchased from J&K Scientific (Beijing, China). *N*,*N*-dimethyl formamide (DMF) was purchased from Tianjin Commie Chemical Reagent Co., Ltd. (Tianjin, China). and tetrahydrofuran (THF) was obtained from Tianjin Yongda Chemical Reagent Co., Ltd. (Tianjin, China). Methylbenzene was purchased from Yantai Chemical Industry Co., Ltd. (Yantai, China) and purified by distillation under reduced pressure. 

### 2.2. Preparation of Tung-Oil-Based Polyurethane (TBPU)

Tung-oil-based polyurethane, TBPU, was prepared via a two-step method. An amount of 5 g of tung-oil-based polyol (hydroxyl number is 282 mg KOH/g) and 15 g PEG (M_w_ ≈ 2000) were added to a four-neck round-bottom flask and phosphoric acid was added to create an acidic medium. Then the mixture was stirred for 2 h at 120 °C in vacuum. Next, the vacuum valve was disconnected, and the flask was filled with argon to prevent moisture. When the temperature dropped to 70 °C, 11.425 g MDI was added to the flask, obtaining an equivalent molar ratio of isocyanate to hydroxyl groups. Afterwards, the mixture was left to react for 2 h at 80 °C to allow prepolymer formation. Anhydrous toluene was added to dilute and regulate the prepolymer at a solid content of 40%. The mixture was cooled to 60 °C and then BDO was used as a chain extender. The flask was heated to 80 °C to react for 2.0 to 2.5 h to further improve the molecular weight.

For reference purposes, polyurethane without tung-oil-based polyol (PU) was prepared by using the same protocol as described for TBPU.

### 2.3. Preparation of TBPU-GO Hybrid Membrane

GO was prepared from natural graphite powders based on an improved method [12,13]. Specifically, 3.0 g of graphite powder was oxidized by 18 g KMnO_4_, in a mixture of 360 mL H_2_SO_4_ (98%) and 40 mL H_3_PO_4_ (85%) under 50 °C for 22 h, and then the resulting pasty mixture was poured into ice-water (1200 mL) and H_2_O_2_ (30 wt %, 20 mL). Then the yellow suspension solution was sonicated and centrifuged for 10 min to collect the GO suspension. Finally, the GO solids went through three cycles of resuspension-centrifugation sequentially with water, HCl (30%), and ethanol in order to completely remove the byproducts and materials. The derived GO was dewatered by vacuum drying at 50 °C.

**Figure 1:**
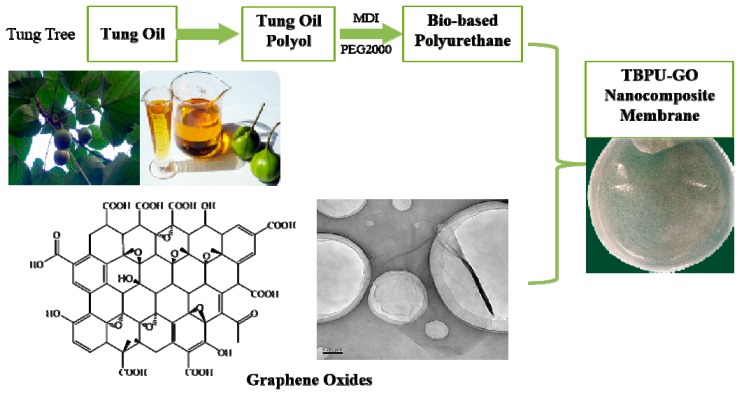
The schematic representation of the GO/TBPU nanocomposite membrane.

The nanocomposite membrane was prepared by solution casting and solvent evaporating (Figure 1). Using DMF as a solvent, TBPU and GO were blended at a GO content of 0.1%, 0.3%, 0.5%, 1%, 1.5%, 2.0%, 5%, and 10% by weight through the following steps: First, the abovementioned GO was dispersed in 10 mL DMF, then the TBPU was dissolved in the mixture to form the blend solution, which was magnetically agitated for 12 h. After that, the casting solution was poured into a polytetrafluoroethylene disk and dried for 24 h at 60 °C in ambient air and reduced pressure. TBPU-GO nanocomposite membranes with a diameter of 80 mm and a thickness of about 0.1 mm were obtained. Pure TBPU membrane was prepared for reference use only.

### 2.4. Characterization

FTIR characterization of TBPU was performed using a Fourier-transform infrared spectrometer (Thereto Nicolet IR200, Thermo Nicolet, Waltham, MA, USA) within the frequency range of 4000 and 400 cm^−1^. The specific heat capacity and glass transition temperatures of the GO/TBPU hybrid membranes and PU membrane were tested using a differential scanning calorimeter (DSC, Perkin-Elmer, Waltham, MA, USA), heated from −70 °C to 450 °C at 10 °C/min under an N_2_ flow rate of 20 mL/min. The mechanical properties of the nanocomposite membranes were measured by a universal testing machine (WDW200D, Shanghai Hualong, Shanghai, China) using rectangular specimen (40 mm × 10 mm × 0.1 mm), and each sample ran for three repeats. The surface and cross-sectional micro-morphology of the nanocomposite membranes were observed by scanning electron microscopy (SEM, S-4700, Hitachi, Hongkong, China). The gas permeability and selectivity were tested by a gas permeation device. The CO_2_ permeability was tested with different transmembrane pressures ranging from 0.1 to 0.5 MPa at room temperature. The permeate flow rate was measured by a soap film gas flow meter. The gas permeability (P, Barrer) and ideal selectivity (α) can be calculated by the following equation,

$$P=\frac{ql}{A\Delta P}$$

in which q stands for gas flow rate (mL/s), l is the membrane thickness (cm), Δp is the constant transmembrane pressure (cmHg), and A represents the effective membrane area (cm^2^). The ideal selectivity is defined as the permeability of CO_2_ relative to that of N_2_, which can be expressed by:

$$\alpha_{CO_{2}/N_{2}}=\frac{P_{CO_{2}}}{P_{N_{2}}}$$

## 3. Results and Discussion

**Figure 2:**
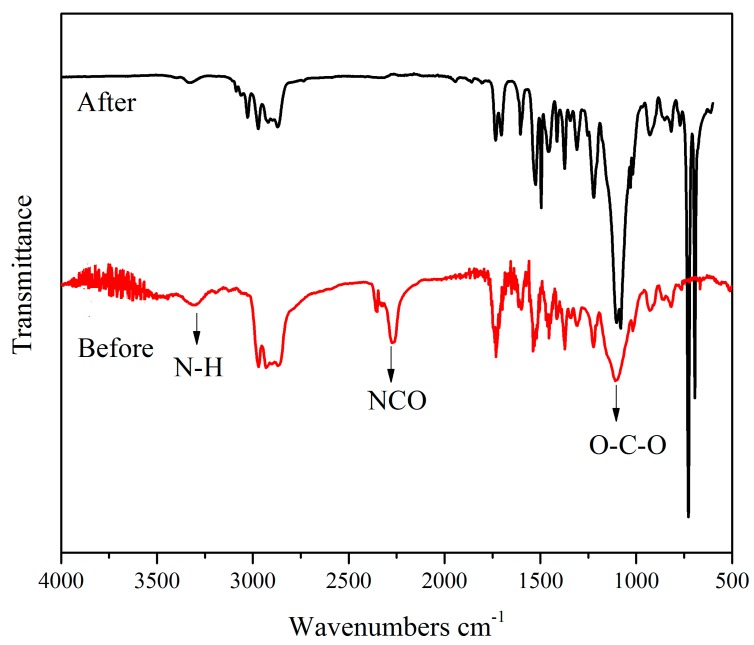
FTIR spectra of tung-oil-based polyurethane before (reactant mixture) and after the synthesis.

FTIR spectra of tung-oil-based polyurethane before (mixture) and after the synthesis are shown in Figure 2. It shows no absorption peak of NCO groups at 2260 cm^−1^, which indicates that the condensation polymerization had completed. The peaks at 3334 cm^−1^ corresponding to N-H reveal that the -NCO of the isocyanate reacted with the -OH to form -NH-COO-. The absorption peak of the free C=O that did not form the hydrogen bond with N-H was at 1740 cm^−1^, while the absorption peak of the C=O that formed the hydrogen bond with N-H was at about 1700 cm^−1^. The absorption peak of O-C-O at 1108 cm^−1^ is attributed to polyether polyol.

**Figure 3:**
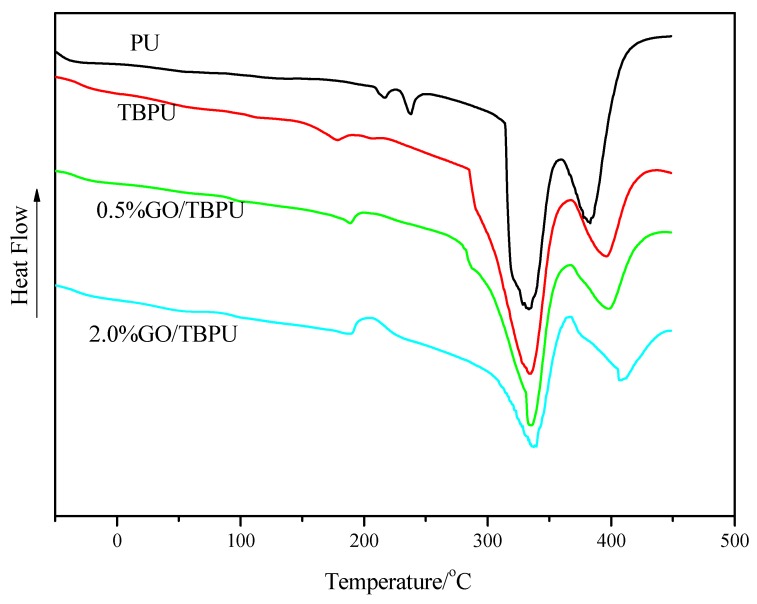
Comparison between DSC thermograms of PU, TBPU, and GO-TBPU.

**Figure 4:**
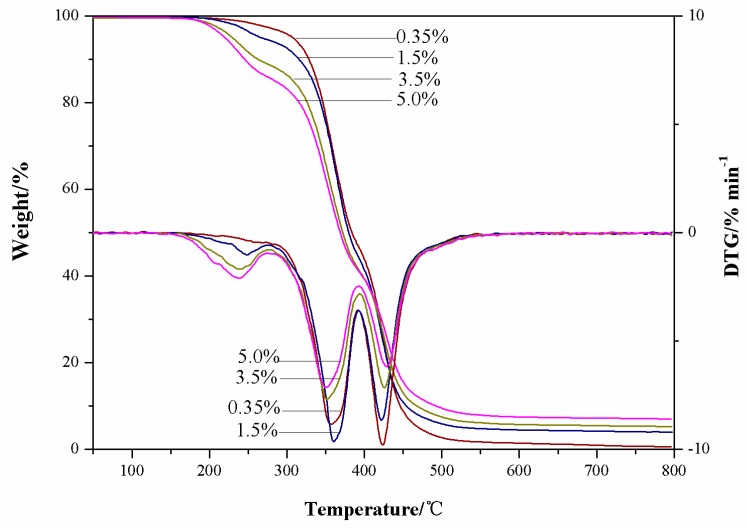
TGA thermograms and derivatives of 0.35%, 1.5%, 3.5%, and 5.0% GO-TBPU in nitrogen.

The thermal analysis DSC diagram of PU, TBPU, and GO-TBPU with a GO content of 0.5% and 2.0% are expressed in Figure 3. The DSC curve clearly shows that the decomposition temperature of TBPU was higher than that of PU. Furthermore, the decomposition temperature increased with the increase of the GO content (TBPU being 396 °C, 0.5% GO-TBPU being 398 °C, and 2.0% GO-TBPU being 406 °C), and the thermal stability of the hybrid film increased, which was consistent with the decomposition temperature of TBPU and 2.0% GO-TBPU in the TGA weight loss curve in Figure 4 [14]. The TGA thermograms and derivatives of GO-TBPU nanocomposite membranes are shown in Figure 4. It demonstrates that the nanocomposite membrane with higher GO content started to decompose faster between 200 and 450 °C, with maximum decompositions around 360 and 420 °C. As the temperature increased to 800 °C, nanocomposite with a higher GO loading retained more residues. The decreasing decomposition temperature of the nanocomposite membrane with the increase of GO is consistent with the thermal properties of GO.

**Figure 5:**
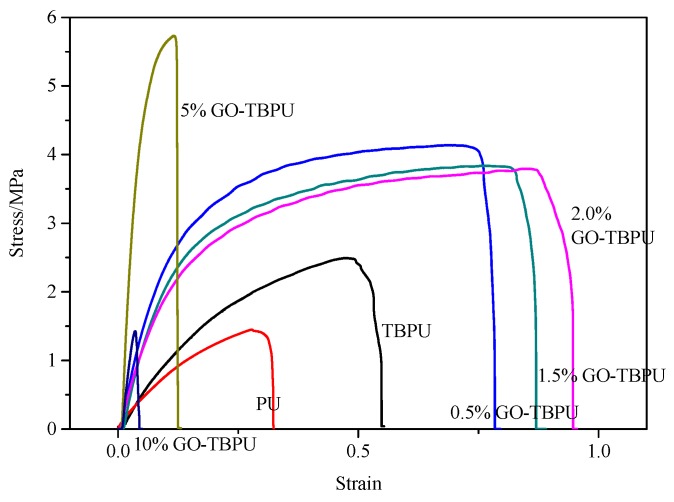
The stress-strain curves of PU, TBPU, and GO-TBPU membranes.

**Figure 6:**
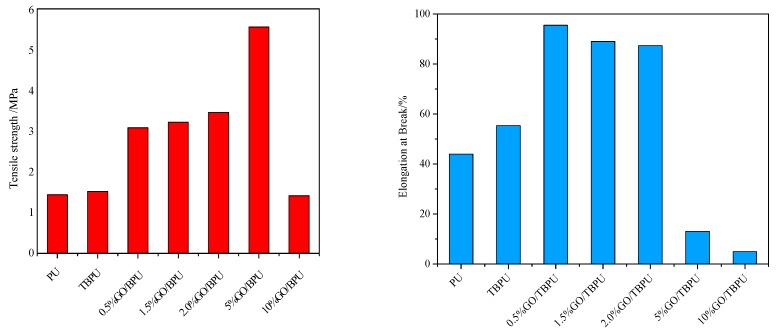
Tensile strength (**left**) and elongation at break (**right**) of PU, TBPU, and GO-TBPU.

The stress strain diagrams of PU, TBPU, and different contents of GO-TBPU nanocomposite membrane are shown in Figure 5, and the corresponding tensile strength and fracture elongation bar graphs are shown in Figure 6. Figure 5 shows that at the same tensile rate of 0.05 mm/s, the maximum displacement and stress of PU (9.0297 mm, 1.2754 N) were lower than that of TBPU (10.9656 mm, 2.244 N). Furthermore, the tensile strength and breaking elongation rate of PU were lower than that of TBPU because the main chain structure of tung oil polyols was short and the molecular weight was relatively low. Additionally, the content of the hard segment of TBPU was higher than that of PU, and, consequently, its tensile strength was larger. Furthermore, TBPU had a better elasticity because of the long side chains in tung oil polyols.

After the additions of 0.5% and 2.0% of GO, the tensile strength and the fracture elongation ratio of the GO-TBPU membrane were about twice as high as those of pure TBPU nanocomposite membrane. That is to say, the mechanical properties of the membranes were significantly improved [15]. With the increase of GO content (e.g., 5%), the tensile strength increased, and the fracture elongation decreased slightly because of the brittle GO.

When the content of GO in GO-TBPU was 5%, the tensile strength greatly increased but the breaking elongation dropped substantially to even less than half of that of the pure TBPU. This means the addition of GO had affected the mechanical performance of the membrane. When the content of GO was increased to 10%, the tensile strength of the film and the elongation of the fracture were reduced. At that point, the brittleness fracture occurred, and the nature of the inorganic substance was completely presented. Based on the comparison, the mechanical properties of GO-TBPU nanocomposite membranes with a GO content of 0.5% to 2.0% were relatively good and suitable [16].

**Figure 7:**
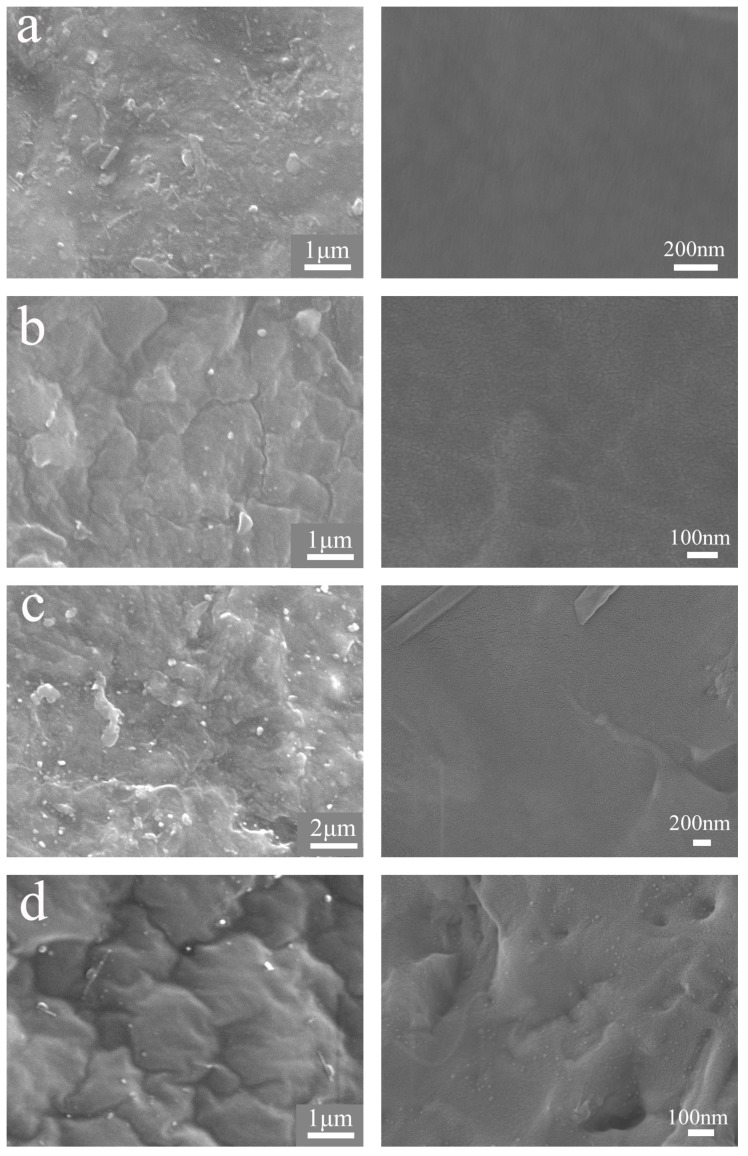
SEM images of the surface (**left**) and cross-sectional (**right**) morphologies of 0.15% GO-TBPU (**a**), 0.35% GO-TBPU (**b**), 1.5% GO-TBPU (**c**), and 5% GO-TBPU (**d**).

The cross section area of GPO-TBPU nanocomposite membrane is mostly uniform and transparent, and the membranes gradually become darker and coarser with increasing GO concentrations. In addition, some agglomerates are observed in membranes when the GO loading is relatively high (Figure 7c). The surface of nanocomposites is smooth, without any agglomerations, and no defect or fracture is detected within 0.35% GO loading. In contrast, morphological changes are observed after introducing the GO into the TBPU: When graphene oxide accounted for 1.5%, there was a small amount of agglomeration in the tung-oil-based polyurethane (TBPU) matrix membrane. The GO sheets stack randomly within the polymeric matrix, which could provide defects such as gas pathways [17].

**Figure 8:**
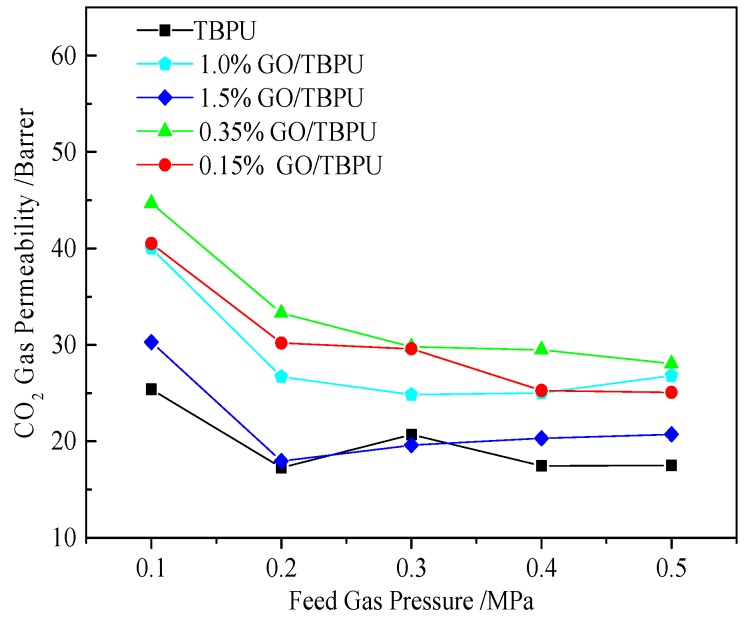
CO_2_ permeability of different GO-TBPU membranes under different pressures.

**Figure 9:**
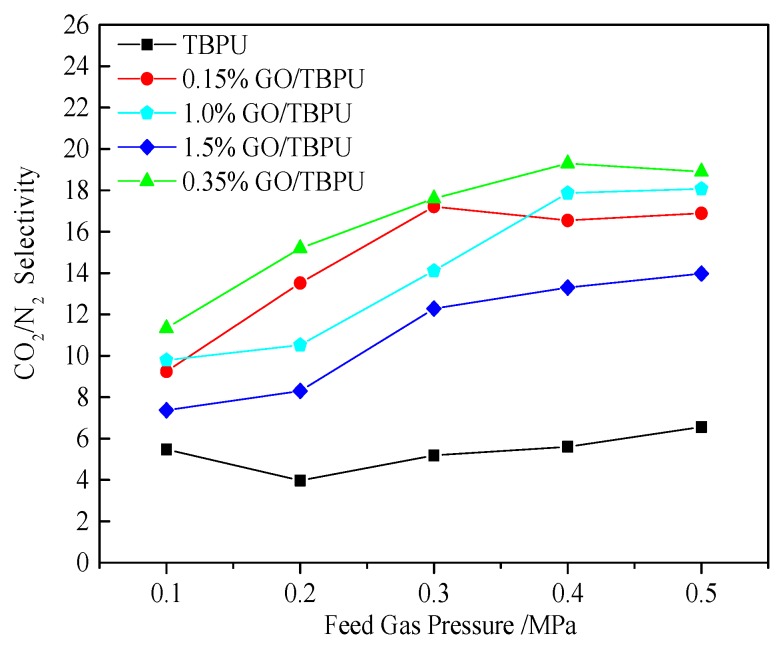
CO_2_/N_2_ selectivity of nanocomposite membranes under different pressures.

Figure 8 presents the penetration curve of the GO/TBPU membrane with different GO contents under different pressures of CO_2_. Figure 9 shows the CO_2_/N_2_ selectivity curve of the GO-TBPU nanocomposite membranes with different GO contents under varying pressures. With the increase of inlet pressure, the CO_2_ permeability began to decline and then stabilized over 0.2 MPa. The permeability of CO_2_ and the selectivity of CO_2_/N_2_ of GO-TBPU nanocomposite membranes were higher than that of the TBPU membrane. With the content of graphite oxide increasing, the CO_2_ permeability and CO_2_/N_2_ selectivity increased at first and then decreased. Due to a low GO content in the TBPU distribution, the two-dimensional structure was helpful for the adsorption of CO_2_, and the conjugated effect forms a common structure with CO_2_ to improve the N_2_ adsorption. At the same time, the addition of GO in hybrid membrane created some interface voids, which could increase CO_2_ permeability. On the other hand, with the content of GO increasing, agglomeration occurred, leading to a reduced performance of the hybrid membrane and a membrane defect structure. Furthermore, the reduction of the conjugated bond had an adverse impact on the separation and permeation of gases.

## 4. Conclusions

In this study, we replaced petrochemical polyols by adding tung-oil-based polyether polyol, and a two-step method was used to synthesize TBPU. Graphene oxide was mixed with TBPU to form a nanocomposite membrane. Then the properties and performances were characterized. The results show that the addition of tung-oil-based polyurethane improved thermal stability; the thermal stability of the hybrid membrane was enhanced when graphene oxide was added. The mechanical properties improved when the content of GO was between 0.5% and 2.0%, with the tensile strength and elongation increasing twofold, but a higher content worsened its performance. The addition of GO created a polyurethane hybrid film with a better hydrophilia compared with pure TBPU. The CO_2_/N_2_ gas separation selectivity was improved as GO improved, with the optimal performance observed at 0.35% GO loading. A higher content of GO worsened the penetration and gas separation characteristics.
